# Non-thermal Technologies for Food Processing

**DOI:** 10.3389/fnut.2021.657090

**Published:** 2021-06-08

**Authors:** Harsh Bhaskar Jadhav, Uday S. Annapure, Rajendra R. Deshmukh

**Affiliations:** ^1^Department of Food Engineering and Technology, Institute of Chemical Technology, Mumbai, India; ^2^Department of Physics, Institute of Chemical Technology, Mumbai, India

**Keywords:** food preservation, pulse electric field, ultrasound, cold plasma, microwave, high pressure processing, irradiation

## Abstract

Food is subjected to various thermal treatments during processes to enhance its shelf-life. But these thermal treatments may result in deterioration of the nutritional and sensory qualities of food. With the change in the lifestyle of people around the globe, their food needs have changed as well. Today's consumer demand is for clean and safe food without compromising the nutritional and sensory qualities of food. This directed the attention of food professionals toward the development of non-thermal technologies that are green, safe, and environment-friendly. In non-thermal processing, food is processed at near room temperature, so there is no damage to food because heat-sensitive nutritious materials are intact in the food, contrary to thermal processing of food. These non-thermal technologies can be utilized for treating all kinds of food like fruits, vegetables, pulses, spices, meat, fish, etc. Non-thermal technologies have emerged largely in the last few decades in food sector.

## Introduction

Food quality is a great concern when processing food for preservation. Conventional food preservation processes expose food to a very high temperature, which no doubt reduces the contamination or microbial load from food, but it also results in some undesirable changes in food, such as loss of nutritional components that are temperature-sensitive, change in the texture of food due to heat, and changes in the organoleptic characteristics of food (1). In thermal processing, food is exposed to heat for a long duration of time, which causes observable changes in food and results in the production of low-grade food (2, 3). The thermal techniques used for preservation result in the formation of chemical toxicants in food that are carcinogenic and harm the human body (4, 5). The amount and the type of toxicants formed also depend on the type of thermal method used for cooking food. Microwave cooking and deep fat frying result in the formation of heterocyclic aromatic amines, which can even cause mutagenic changes in the body (5, 6). Thermal treatment can also cause loss of water from food, oxidation of lipids, and changes in the composition of fatty acids. Barbequing of meat causes loss of meat juices that mainly contain saturated lipids stored in the form of adipose tissue, leading to a decrease in saturated fatty acid and an increase in polyunsaturated fatty acid in the final product. The presence of polyunsaturated fatty acid makes the final product more susceptible to lipid oxidation and decreases the quality of product, imparting an off-flavor with a reduced mouthfeel (7). But now, consumers' awareness regarding food safety has increased and they demand food free from microorganism and with high nutritional qualities and excellent mouthfeel. This led food professionals to search for a better alternative, like non-thermal treatments. In non-thermal processing, food is exposed to ambient temperature for a very limited period of time, i.e., for ~l min or less, which causes no change in the nutritional composition of food, the texture remains intact, and the mouthfeel is not lost (8–10). The rise in consumer demand for fresh food with longer shelf-life and good sensory qualities led to extensive research in the field of non-thermal treatment of food (11). Thermal technologies that require huge energy consumption and produce low-grade food can be fully or partly replaced by the consumer-, environment-, and pocket-friendly (since they are economical) non-thermal technologies for food processing and preservation (12–14). Various non-thermal food processing treatments came into light since the last few decades, which included pulsed electric field, cold plasma, ultrasonication, microwave, supercritical technology, etc. These non-thermal treatments unmask food to treatment conditions for a fraction of seconds, which results in the reduction of the microbial load in food with an increase in shelf-life, with good sensory and textural characteristics (15, 16). The preservation effect of non-thermal technologies is more than that of thermal technologies because there is no chance for the formation of any undesirable products/by-products in food or on the surface of food since it is not exposed to higher temperatures (17). Pulsed electric field is an extensively used non-thermal processing treatment in the food sector. It is mostly exploited for liquid food including fruit juices, alcoholic beverages, non-alcoholic beverages, etc. It can be directly applied on the entire fruit. It damages the cell wall of microorganisms, leading to the death of microbes and the reduction of the microbial load (18). The intensity of pulse and pulse width play important roles in microbial reduction in food exposed to pulse electric field treatment (19). Non-thermal treatment can also arrest the activity of enzymes, leading to the spoilage of fruits and vegetables. Cold plasma technology is extensively used to enhance the physiological properties of proteins and carbohydrates in food, so that they can be used in numerous applications in food processing. Gaseous cold plasma processing has been used for improving the cooking and textural properties of food grains (20, 21). It also inactivates the microbes present on the surface of the food product. Cold plasma treatment time plays an important role in achieving the desired results (22–24). Ultrasonication is an energy-efficient non-thermal treatment usually used for the intensification of processes like synthesis, extraction, and preservation of food and allied products. Ultrasonication duty cycle and exposure time have positive effects on food. A perfect combination of duty cycle and exposure time can be utilized in developing safe and nutritious food with ultrasonication (25, 26). Other technologies such as ultra-pressure treatment and irradiation are also exploited in the food processing sector to achieve food safety with minimal or no loss of the nutritional, textural, and organoleptic characteristics of food (27, 28). These non-thermal treatments result in a decrease of the microbial load by altering the structure of the membranes in bacterial cells and unfolding of the helical structure of the DNA of the genetic material of microbial cells, leading to the death of microbial cells in a short period of time. Apart from the reduction of the microbial load, these non-thermal treatments are also used for the extraction of bioactives from plant and animal sources having nutraceutical food application for the intensified synthesis of the nutraceutical components, dehydration, for enhancing the physical and chemical properties of food constituents, etc. (29–35). In spite of the many advantages of these non-thermal technologies in the food sector, they are rarely used in food industries and remain at laboratory scale only. There is a great need for the understanding of the construction and workings of these non-thermal technologies and their action on food. There is enough scientific literature available on these technologies. The present review focuses on the recent status of non-thermal techniques in food processing industries to enhance the quality of food products, the effects of these non-thermal techniques on food components, instrumentation used for these non-thermal techniques with a focus on the limitations of these techniques for large-scale production and how they could be overcome, and the future prospects of these techniques in food processing industries. This comprehensive review will definitely help food scientists and technologists working in the field of non-thermal technology since non-thermal treatment is gaining research interest due to its numerous merits over thermal techniques.

## Non-Thermal Technologies

### Ultrasonication

#### Theory

Ultrasonication is an emerging non-thermal technology in the food sector, but it is already a well-established technique in other processing sectors (36). In simple words, ultrasound is a sound wave bearing certain frequency that is more than the normal human hearing frequency, i.e., above 20 kHz (37). When ultrasonic waves oscillate through the medium, they generate many expansion and compression effects in the medium. There is a formations of small cavities due to the presence of air. The cavities formed grow to a desired size and then collapse. When these cavities collapse, they generate both a large amount of energy and local hot spots, and thus there is an increase in heat and mass transfer rates (38). Ultrasonication is used to speed up the chemical synthesis of organic compounds and increase the yield of reaction because the ultrasonication effect results in enhanced heat and mass transfer. Ultrasonication is used with different frequencies, which are classified as low-frequency, medium-frequency, and high-frequency ultrasonication, with frequency ranges of 20 kHz–100 kHz, 100 kHz−1 MHz, and 1 MHz–100 MHz, respectively (39). Low-frequency ultrasonication produces large shear forces in the medium, whereas high-frequency ultrasonication produces less shear forces in the medium. The medium frequency results in the formation of radical species, and this frequency range is considered to be optimum for various sonochemical-assisted process, but the formation of chemical radicals can bring about undesirable changes in food, such as oxidative changes in lipids and proteins (40). Ultrasonication is done using an ultrasonic horn, which is dipped in the liquid solution or juice and is treated with certain treatment frequency. Ultrasonication can be done using an ultrasonic bath, in which the food material or packaged food is kept and the sound waves are generated in a bath that creates ultrasound effect and brings about desired changes in food (41).

#### Applications

In food processing, frequency in the range of 20 kHz–100 kHz is used for the extraction of bioactives, emulsification, cooking, debittering, intensified synthesis, etc. Jadhav et al. (42) reported on the synthesis of designer lipids using sonication as an excellent alternative for intensified yield. The authors reported a maximum yield of 92% in 6 h of reaction. Due to the increase in energy, there is a generation of high-energy spots that increase the rate of mass transfer, and the reaction is completed in a shorter period of time. Ultrasonication-assisted synthesis is rapid compared to the conventional synthesis process (43). Ultrasound also assists the interfacial transfer of molecules, which enhances the efficiency of the process of extraction of bioactives from plant and animal sources. The extraction process not only increases the yield of the extraction process but also improves the physical and chemical properties of the extracted compound. One such recent study by Sun et al. (44) reported that protein extracted using ultrasonication showed superior properties in terms of the size of the particle, emulsification power, and structure. Ultrasonication-extracted particles with small particle size and larger α helix structure have improved emulsifying power when treated with sonication for 30 min at 20 kHz. Cheila et al. (45) designated ultrasonication as a greener approach for the extraction of bioactives from the leaves of velame. Ultrasound increased the yield of extracted bioactives to 94% in 39.5 min using indirect ultrasonication. Ultrasound has been proven to be an intensified extraction process for the extraction of oil from olive fruit, soybean, and flaxseed (46, 47). It is also employed for the extraction of bioactives from different parts of plants, fruits, and vegetables (48–51). Ultrasonic-assisted filtration process is also very effective and is of importance to dairy and beverage industries. In cheese making, the membrane filtration process is used for the complete separation of milk protein from other milk solids (52). Ultrasonication also aids in the processes of freezing, drying, and thawing of food products (53–55). Mothibe et al. (56) used ultrasonication as a preliminary processing step before the dehydration of apples and reported that the drying time was reduced and the dried apple had a good texture with less water activity. The authors reported that treatment at 25 kHz and time of 15 min showed good results. As the treatment time increased, there was more loss of soluble solids from apple. The ultrasound-assisted process not only decreases the drying time but also enhances and retains the texture after rehydration. Rehydration refers to the absorption of moisture by the dried food (57). Tao et al. (58) showed that ultrasound-rehydrated white cabbage showed a higher rate of rehydration compared to the untreated sample. Similar studies were reported for the rehydration of carrot and green pepper (59, 60). It is also used as a pretreatment for convective drying and freeze drying (61, 62). Ultrasonication is also effective for the preservation of food products by using brine solution. Carcel et al. (63) reported on the use of ultrasound in the treatment of pork loin with a brine solution; ultrasound was applied to this solution. The authors reported that the ultrasound-assisted brine sample has more concentration of brine in it with good color and texture of pieces of pork loin compared to the untreated sample. Ultrasonication is also beneficial for the process of degassing in carbonated beverages and is a good replacement for the processes of pasteurization and sterilization in the reduction of microbial load in food and food products (26). Ultrasound has successfully proven its potential in the food sector in various critical areas like food preservation, extraction, intensified synthesis, and improvement of the physical and chemical properties of food. The very limited technical information about ultrasonication and consumer awareness about ultrasonic-processed food have been the hindrance in the commercialization of this process in food industries. However, the treatment must be studied on bulk food to understand its effect so that it can be implemented at industrial scale.

### Cold Plasma Technology

#### Theory

Plasma is the fourth state of matter after solid, liquid, and gas. The term plasma was used by Langmuir in the year 1925 (64). An increase in the kinetic energy of solids leads to the heating up of molecules, and there is phase transformation from solid to liquid, further increasing the energy of liquid and converting liquid to gas. The increase in energy causes disintegration in the intermolecular structure. When the energy of gases crosses a certain value, it results in the ionization of gas molecules (65). Ionization of gas molecules gives rise to plasma. Hence, it is known as the fourth state of matter. Basically, plasma treatment is divided into two types: thermal plasma and cold plasma (non-thermal). Thermal plasma produces huge energy by utilizing high temperature. Cold plasma is a non-thermal treatment that works in the temperature range 25–65°C (66). When gas is ionized, free radicals (ions, electrons, etc.) are formed. The composition of the plasma reactive species largely depends on the composition of gas which is ionized (67). The gases commonly used for the generation of plasma include argon, helium, oxygen, nitrogen, and air (68). These gases are subjected to any of the types of energy like thermal, electrical, magnetic field, etc., to generate plasma containing positive ions, negative ions, and reactive species like ozone and singlet oxygen (O) (69). Based on the nature of plasma, it has found various applications in the fields of chemistry, chemical engineering, textile, electronics, surface coating, and pharmaceuticals and in food sectors (70). In the food sector, cold plasma can be used for the reduction of the microbial load in food or on the surface of food, enhancing the physical and chemical properties of food constituents like lipids and proteins, and for the sterilization of food processing equipment, inactivation of food spoilage enzymes, treatment of food packaging material, and treatment of wastewater (71). Cold plasma is produced at near ambient temperature and does not depend on high temperature for microbial inactivation. Since the temperature used is ambient, there are no chances of thermal damage to heat-sensitive food material (16).

#### Applications

Microbial inactivation in cold plasma is due to the effect of reactive species on the microbial cell. Reactive species damage the DNA of cells, induce oxidation in protein, and damage the cellular components of microbes, causing cell death (72). Lin et al. (73) have reported that cold nitrogen plasma shows inhibitory action on *Salmonella enterica* serovar Typhimurium biofilms formed on the outer surface of an egg shell. The sample was treated at 600 W for 2 min, which reduced the catabolic and anabolic activities of the *S. enterica* serovar Typhimurium by 82.2%. Devi et al. (74) showed 97.9% and 99.3% reductions in the growth of fungal species such as *Aspergillus parasiticus* and *Aspergillus flavus*, respectively, on the ground nut surface when treated at 60 W plasma power. In the food sector, atmospheric pressure cold plasma is used in combination with other gases like helium, argon, etc. Recently, Bang et al. (75) reported on the combination of antimicrobial washing and in-package cold plasma treatment to mandarin oranges for reduction of the microbial load. Treatment at 26 and 27 kV for 1–4 min inactivated *Penicillium digitatum*. The combined effect of washing with an antimicrobial solution and cold plasma treatment reduced the load of *P. digitatum* in the package without affecting the texture, sensory, and nutritional qualities of the oranges. The treated oranges showed a decrease in ripening damage compared to the untreated oranges. Liao et al. (76) reported the use of cold atmospheric pressure-activated water or plasma-activated ice as a cold storage medium for seafood. Shrimps stored in plasma-activated water showed longer shelf-life due to bacterial inactivation, and there was no observable change in the texture of shrimps. The total volatile base nitrogen value for shrimps stored in plasma-treated ice was lower than 20 mg/100 g on the ninth day, which was higher than the 30 mg/100 g for shrimps stored in untreated water or ice. Cold plasma treatment is also effective against the pathogenic microbes present in food and processed food products. One such recent study reported by Gan et al. (77) showed the effectiveness of cold plasma against *Escherichia coli* and *Saccharomyces cerevisiae* in the juice of chokeberries. The authors reported that treatment of 4 min decreased the loads of *E. coli* and *S. cerevisiae* by 2.27 and 1.23 log CFU/ml, respectively. The treatment was seen to be more effective against the inactivation of *E. coli* compared to *S. cerevisiae*. Similar studies on the inactivation of *E. coli* were also reported by Shah et al. (78). Cold plasma is also used for disinfection of the surfaces of food processing equipment to remove the microbial load before the processing of food. Hou et al. (79) investigated the effect of atmospheric pressure cold plasma on bacterial inactivation and the quality of blueberry juice. There was a decrease in the load of *Bacillus* spp. in juice with exposure to cold plasma for 6 min by 7.2 log CFU/ml. The short exposure time resulted in good color and bioactive component retention in juice. Similar results were reported for the preservation of fresh tomato juice (80), cloudy apple juice (81), and apple, tomato, orange, sour cheery nectar (82), and whey grape (83) juice. It is also used for the preservation of meat and related products by reducing their microbial load. Roh et al. (84) studied the effect of cold plasma treatment of 3.5 min against pathogenic microbes in chicken breast. The treatment resulted in decreases in the loads of *E. coli* by 3.9 log CFU/g of chicken, *Listeria monocytogenes* by 3.5 log CFU/g, and Tulane virus by 2.2 CFU/g of chicken. Similar results were reported for the inactivation of *Salmonella* in chicken breast (68, 85, 86) and the microbial load in sea snail (87). The technology is also used for enhancing the physical and chemical properties of food constituents (16, 23). This technology also finds application in enhancing the physical and chemical properties of carbohydrates and proteins in order to increase their functionality and application in food. In the recently published research by Jahromi et al. (88), sodium caseinate in granular form was subjected to 10-kHz treatment for 0, 2.5, 5, and 10 min. With the increase in treatment time, the physical and chemical properties were enhanced. The hydrophilicity of protein increased due to unfolding of the protein structure. Water solubility increased from 20.6 to 30.28%. Tensile strength increased from 5.04 to 7.17 MPa for the 10-min treatment and decreased to 4.73 MPa at 15 min. The effect of cold plasma especially on milk protein is reported by Sharma et al. (89). Cold plasma contains various reactive species, and it is found that these reactive species may trigger the process of lipid oxidation during storage. Gao et al. (90) reported that cold plasma treatment at 70 kV for 180 s triggers the oxidation of lipids during storage. The thiobarbituric acid-reactive substance (TBARS) value increased to 2.48 from 1.43 mg MDA/kg when stored at refrigeration temperature for 5 days, which was 0.37 mg MDA/kg for the control sample of chicken patties. In the TBARS assay, malondialdehyde (MDA) is measured. MDA is a by-product resulting from the process of lipid peroxidation. This MDA reacts with thiobarbituric acid and forms a pink chromogen, known as TBARS. This oxidative degradation of lipids in food can be controlled by altering the treatment conditions, such as the exposure of food to plasma for a short duration of time or the addition of antioxidants in food to overcome these disadvantages of cold plasma on lipids in food. Food containing higher lipid levels can be exposed for a shorter time to cold plasma compared to food with a low lipid content (91, 92).

### Supercritical Technology

#### Theory

Supercritical technology makes use of supercritical fluids, which are considered as a good replacement for organic solvents used in various operations (93). When a fluid is heated beyond its critical temperature and critical pressure, it attains a supercritical state and is referred to as a supercritical fluid. The supercritical fluid shows some properties of gas and some properties of liquid. It shows density like liquids and diffusivity and viscosity like gas (35). Supercritical fluid shows enhanced properties similar to liquid, and hence it can be used as a solvent with an increased rate of mass transfer during the extraction of bioactives from various plant and animal sources. The properties of fluids can be altered with changes in temperature and pressure. Many fluids are used for supercritical operations, but carbon dioxide finds special attention as an excellent supercritical fluid in the food processing sector because it can achieve a supercritical state at a modest temperature and pressure (31.1°C and 7.4 MPa, respectively). Supercritical fluids are extensively used in food industries for extraction, microbial inactivation, enhancement of mass transfer in synthesis, etc. Among all the applications, the supercritical technology is extensively employed for extraction purposes.

#### Applications

Supercritical carbon dioxide is used for the purpose of extraction since it is non-toxic and can be separated from the final product without much effort (94). Natural bioactives that are extracted are sensitive to temperature and oxygen. In the presence of carbon dioxide, the supercritical extraction temperature is very low and there is no chance of the presence of oxygen; hence, the quality of the extracted material is high and can be used as a functional ingredient in various nutraceutical formulations. Recent studies reported by Lefebvre et al. (95) showed that supercritical carbon dioxide is effectively utilized as an excellent tool for the selective extraction of antioxidants from rosemary. The temperature and pressure of CO_2_ were 25°C and 20 MPa, respectively, which were ideal and did not affect the purity of the extracted products. Santos et al. (96) investigated the extraction of bioactives from feijoa leaves using supercritical and pressurized liquid extraction. The authors reported that pressurized extraction gave more yield of antioxidant and antibacterial components, but these extracted components were not effective in their function, while supercritical extraction of antioxidant and antibacterial components at 55°C and 30 MPa showed higher effectiveness against pathogenic bacteria including *E. coli*. The technique is also used for the extraction of functional and nutraceutical ingredients from microalgae (97), oil from fruit seeds (98–101), oil from olives (102), oil from ginger (103), extraction of corn germ oil and green coffee oil (104, 105), essential oil extraction (106, 107), and extraction of bioactives such as carotenoids, lycopene, astaxanthin, anthocyanins, and quercetin (108–110), which can be used as components in nutraceutical formulations. Extraction using supercritical carbon dioxide has been common for many years in the food processing industry. Apart from this, supercritical technology is also used for reducing the microbial load in food. Since the operating temperature in supercritical treatment is low, the original characteristics of food, along with its organoleptic characteristics, is retained (111). Supercritical fluid treatment reduces the pH of bacterial cell, which leads to the rupture or bursting of cells and the inactivation of bacterial enzymes that are responsible for catabolism and anabolism; thus, the bacterial cell dies and reduces the load of microbes in food and related products (112). It is extensively used for the preservation of fresh agricultural products including fruits, vegetables, and their juices (113). Bertolini et al. (114) studied the effect of supercritical carbon dioxide on the decrease of microbial load in pomegranate juice and compared it to traditional pasteurization and high-pressure processing. The authors reported that supercritical-treated juice showed bacterial growth below the detection level after storage for 28 days. The total phenolic content increased by 22%, but it decreased in traditional pasteurization by 15%. The antioxidant activity of the phenolic components was more in supercritical-treated juice compared to that in high-pressure processing and traditional pasteurization. Similar results were reported for the preservation of coconut water (115), sports drink (116), and liquid food (117). Supercritical fluids are also used for the preservation of ground meat. Yu and Iwahashi (118) treated ground beef with high-pressure carbon dioxide at 1 MPa pressure for 26 h and found a reduction in the microbial load. The critical review of the literature showed that the supercritical technology has bright prospects in the food processing sector not only for extraction but also for the preservation and enhancement of the physiological properties of food constituents to be used as functional ingredients in functional and nutraceutical formulations.

### Irradiation

#### Theory

Gamma rays with high energy, X-rays, and high-speed electrons are approved irradiations to be used in food processing industries. Radionuclide ^60^CO and ^137^Cs producing gamma rays are used for the production of elevated energy photons. X-rays with energies up to 5 MeV are used in the food processing sector. High-speed electrons with energy of 10 MeV are used in food industries for various applications (119). Irradiation effects are achieved without an increase in the temperature of food. Since the temperature of food is not raised, there is no chance of damage to the components in food that are sensitive to heat (120). The penetration ability of a high-speed 10-MeV electron is up to 39 mm deep in food with high moisture content. X-rays and gamma rays can reach deep into the food material (119, 121). These radiations result in the unfolding of DNA and damage to the nucleic acid, and the ionization of water molecules results in oxidative damage to the microbial cells; thus, there is reduction in the microbial load of food (122).

#### Applications

Irradiation is mostly employed in the food processing sector for the preservation of food products. It is effective against pathogenic microbes including *E. coli, Staphylococcus*, and *Salmonella* (123, 124). Changing the intensity of irradiations shows more intense effects on the inactivation of microbes in food. Irradiation is also used in the preservation of meat for several days. Ready-to-cook chicken stored for 15 days treated with gamma radiations of intensities 0, 1.5, 3, and 4.5 kGy showed excellent result for the inactivation of *L. monocytogenes, E. coli*, and *Salmonella typhimurium*, with *D*_10_ values of 0.680, 0.397, and 0.601, respectively. The ready-to-eat chicken showed good sensory and textural characteristics even after 15 days of storage (125). Irradiation technology also enhances keeping qualities of food and keeping food fresh by the inactivation of microbes causing foodborne diseases (126). It has been found that the use of irradiation scan results in some undesirable changes in food if treated at high irradiation doses, mostly seen in food like meat whose color and lipids are the main defining factors and a slight change in color and lipids may lead to rejection by consumers (127). It is also seen in cereals and food grains (128). Thus, to achieve the desired inactivation in food with no or little change in the food composition and processed food products, irradiation is usually done with a low dose, and the irradiation effect is combined with the use of antimicrobial agents (129). Irradiation is successfully used for achieving microbial inactivation, like the microbial load in fresh pasta (130) and for enhancing the physical and chemical properties of food, such as those of wheat (131), garlic bulbs (132), grape juice (133), mangosteen fruit (134), apple juice (135), etc. Despite the many advantages of irradiation technology mostly in food preservation, consumer acceptability of irradiation-processed food is low because of the wrong perception of the word “irradiation.” For a non-food technologist, irradiation is the generation of some carcinogens in food, as the word is similar to “radiation therapy” (122). Low consumer acceptance is the great hindrance in the development of this technology in the food industry. Changing the views of consumers and encouraging them to buy irradiated food could be solutions for the development of this technique, and designing simpler and reliable instrumentation and overcoming the myths about this technology among consumers will greatly influence the market of irradiated food in the coming years.

### Pulsed Electric Field

#### Theory

Pulsed electric field (PEF) is an emerging non-thermal technology and finds various applications in the food sector. The growing demand for safe food with nutritional qualities has influence on the use of pulsed electric field in the food sector. In pulsed electric field, a pulse of high field intensity is applied to food for a very short duration of time (19). Usually, for the treatment of food, the field intensity is from 25 to 85 kV/cm, and the exposure time is a few milliseconds or nanoseconds. Since food is exposed to pulsed electric field for a very short duration of time, there is no chance of heating; hence, undesirable changes in food due to high temperature are eliminated (18). In the early 1950s, PEF was used for preservation by inactivating microbes. Since then, it has developed a lot in recent years and has been widely used for microbial inactivation in food. A typical PEF device consists of a food treatment chamber, a control system, and a pulse generation unit. The food is kept in the treatment chamber in between two electrodes generally made of stainless steel (136). Pulsed electric field is generally used for liquid food or semi-solid food that can flow easily (137). There is a damage to the cell membrane of microbes due to the high field intensity. Hydrogen peroxide is found in a PEF-treated sample, and it brings about oxidative changes in the cell lipids and protein of the bacterial cell, which also inactivates the metabolic enzymes, thus causing cell death (138). The efficiency of PEF in reducing microbial load largely depends on the intensity of field applied, the total exposure time, temperature, and energy.

#### Applications

PEF is extensively employed for increasing the shelf-life of food by decreasing the microbial load. A recently published study by Preetha et al. (139) showed that PEF with an intensity of 5.6 W/cm^2^ was effective against *E. coli* in flowable food like pineapple and orange juice and coconut water, with decreases in the *E. coli* load of 4.5, 4, and 5.3 log CFU/ml juice respectively. Similar results with moderate PEF intensity are also effective for microbial inactivation in fruit juices (140). Microbial cells that are larger in size are exposed to PEF easily, but smaller microbial cells may resist the treatment and remain unaffected (141). Apart from microbial inactivation, PEF is also effective in the deactivation of food spoilage enzymes. Similar studies are reported for the inactivation of enzymes in apple and carrot juice (142) and pine nut (143). López-Gámez et al. (144) investigated PEF treatment of 580 J/kg on carrot, which showed enhanced anabolism for the production of phenolic compounds over a storage period of 36 h. PEF is also extensively used in the extraction of bioactives from many natural sources. A recent study was reported by Käferböck et al. (145) on the extraction of functional components from microalgae using PEF with a frequency of 300 Hz and pulse width of 4–32 μs. The PEF treatment resulted in enhanced extraction of the functional components with high purity, which can be used for their nutraceutical application in food industries without purification. A similar study for extraction from the microalgae *Haematococcus pluvialis* is reported by Gateau et al. (146). Other studies were on extraction from apple peels (147), cyanobacteria (148), tomato (149), and from cinnamon (150). Apart from the known application of PEF in microbial inactivation, extraction, and physiochemical changes, nowadays, PEF is also employed for unit operations like dehydration and freezing. Liu et al. (151) reported on PEF with an intensity of 0.6 kV/cm and exposure time of 0.1 s that resulted in a decrease in the drying time of carrot by 55% at 25°C and 33% at 90°C. The PEF-treated sample showed good textural properties and color after rehydration compared to the untreated sample. PEF pretreatment before the drying operation results in enhancing the vitamin and mineral contents in food (152). It is also used in the freezing of food and improves the quality of food during the thawing process (153). PEF treatment leads to an improvement in the coefficient of diffusion for water before drying, reduces the time required for freezing and drying food, and maintains the quality of rehydrated and thawed food for a longer period. PEF is also employed for enhancing the physical and chemical properties of major food components such as polysaccharides, proteins, etc. (154–156), and for the modification of potato starch (157) and the properties of oat flour (158). PEF also enhances the rate of reaction; because of the high intensity, there is an enhanced heat transfer, which increases mass transfer in the esterification (159) and chelation (160) reactions. The numerous good effects of PEF on food products make it a good non-thermal treatment method. There is a need for the development of high-strength PEF instrumentation for commercial application.

### High Hydrostatic Pressure

#### Theory

High hydrostatic pressure (HHP) utilizes a very common medium, i.e., water, to apply the pressure on the product to be treated. HHP can bring about a significant decimal decrease in the population of pathogenic Gram-negative bacteria, Gram-positive bacteria, yeast, and mold and helps in food preservation for a longer duration. The reduction in microbial load depends on the pressure and temperature during treatment. It largely depends on the type of food processed. Food, when subjected to HHP treatment, undergoes high pressure for a short duration of time. The pressure applied to food during treatment is in the range of 200–700 MPa (161). The quality in terms of nutritional components, sensory, and texture of HHP-processed food is excellent since the food is exposed to treatment conditions for a very short period of time (162). It is found that the HHP treatment is more effective against eukaryotes, Gram-negative bacteria, protozoa, and parasites than yeast and mold, which are inactivated at much higher pressure (163). The instrumentation required for HHP is very simple and easy to operate. It consists of a pressure compartment in which food is kept and water is introduced into the chamber; food is then pressurized using this water (164). Thus, HHP-treated food shows fresh-like attributes since there is no intervention of high temperature and chemical additives. Pressure of 350–450 MPa is sufficient for the inactivation of Gram-negative bacteria, yeast, and mold at room temperature, but to inactivate Gram-positive bacteria, pressure more than 1,100 MPa is required (165). The high pressure results in damage to the cell membrane of microbial cells, which changes the permeability of the microbial cell wall and membranes. The coiled protein structure breaks and there is destruction to microbial cell enzymes, which alter the metabolic pathways; finally, the microbial cell dies, leading to a decrease in the microbial population in food (161).

#### Applications

HHP treatment has been proven to be efficient in the inactivation of microbes in a wide range of food products, including processed fruits, meat and meat products, and dairy products, which serve as an excellent medium for microbial growth. The recently published study by Bulut and Karatzas (166) investigated the effectiveness of HHP against *E. coli* in liquid food. Orange juice was stored at −80°C, and then HHP treatment was applied to the juice with pressure of 250 MPa for 900 s. The HHP treatment reduced the microbial load by 4.88, 4.15, and 4.61 log CFU/ml for orange juice with pH 3.2, 4.5, and 5.8, respectively. Cap et al. (167) investigated a decrease in *Salmonella* spp. load in meat using HHP treatment. The HH pressure of 500 MPa for 60 s was enough to inactivate the *Salmonella* spp. in a chicken breast sample without any effect on the organoleptic and sensory attributes of chicken. Cava et al. (168) showed that dry cured sausage can be preserved for more than 60 days with inactivation of *L. monocytogenes* by 3.2 log CFU/g with HHP treatment of 600 MPa for 480 s. There was no oxidative damage to the lipids and proteins in food up to 60 days. HHP has no effect on the oxidation of lipids; thus, it does not contribute to the development of rancidity in food. de Jesus et al. (169) reported that HHP is not only effective in the reduction of the microbial load but it can also be effectively utilized for the extraction of antioxidant, anthocyanin, and phenolic compounds with various nutraceutical properties in food. Similar studies on extraction were reported from tomato waste (170), pomace of grapes (171), red microalgae (172), egg yolk (173), and from gooseberry juice (110). HHP also enhances the physical and chemical properties of fermented juices and increases bioactives in the fermented juice (174). HHP is also effective in the preservation of human breast milk (175, 176). It is also beneficial for intensifying the technical and functional properties of milk proteins for their increased application in various functional and nutraceutical foods (177). HHP has been the potential treatment not only for bacterial inactivation but also for the extraction and enhancement of antioxidant, phenolic, bioactive, and functional components from various sources, suggesting its application potential in various nutraceutical, pharmaceutical, health, food, and related industries. There are many technical obstacles in building the HHP units that are feasible for the treatment of high-volume food, and hence there are very few or no HHP-treated food available in the market today.

### Pulsed Ultraviolet Technology

#### Theory

Ultraviolet technology is a very economical, non-thermal technology. It is basically used to reduce the microbial load on the surface of food materials that are indirectly exposed to radiation, which are grouped as UV-A in the electromagnetic spectrum in range of 320–400 nm, UV-B in the range of 280–320 nm, and UV-C in the range of 200–280 nm (178). When food is exposed to UV-C, with 200–280 nm, these short wavelengths are absorbed by the microbial cell nucleic acids. These absorbed photons cause the breakage of the bond and interlinking between thymine and pyrimidine of different strands and the formation of dimers of pyrimidine. These dimers prevent DNA transcription and translation, thus leading to the malfunctioning of the genetic material, which causes microbial cell death (179). The photons of UV-A and UV-B result in the destruction of the cellular membranes, proteins of microbial cells, and other cellular organelles, which causes death of the microorganisms present in food (180).

#### Applications

Pulsed UV technology is among the most popular non-thermal technologies in the food processing sector. Owing to its economical nature, it is also being experimented on a pilot scale for the inactivation of microbes. A recent study by Fenoglio et al. (181) on a pilot-scale UV inactivation of pathogenic microbes showed that the intensity of UV-C with 390 mJ/cm^2^ leads to the inactivation of pathogenic bacteria in fruit juices, with log reductions of 6.3 for *Lactobacillus plantarum*, 5.1 log CFU for *E. coli*, and 5.5 for *S. cerevisiae*. Similar studies on the inactivation of microorganisms in fruit juices are reported in apple juice (182), orange juice (183), and cantoloupe melon juice (184). Ultraviolet inactivation is also extensively used for the inactivation of microbes present in milk and milk products (185). Ultraviolet radiation also shows useful effects on the chemical and physical properties of food. Kumar et al. (186) showed that UV-C radiation with 254 nm was able to enhance the physical and chemical properties of protein from wheat. Therefore, it can be used for many applications in food industries. Recent studies have shown that ultraviolet treatment of fresh fruits and vegetables (after harvesting) not only results in microbial inactivation but also increases the antioxidant content and enhances its activity (187). UV treatment is also used for the reduction of toxins in food (188). With many positive effects on food, there are some studies reported in the literature which showed that high-dose UV treatment can lead to a decrease in the color of food and adversely affects the texture of solid food (189). All food products have different textures with uneven and rough surfaces, so the ability of radiation to reach inside the food material may be reduced, decreasing the efficiency of the inactivation process. Thus, to increase the efficiency of the process and achieve higher inactivation, non-thermal processes are usually coupled or antibacterial agents are used along with UV treatment (190, 191). Due to its simple operation, UV is one of the well-established non-thermal processing technologies adopted by food processing industries to produce food with longer shelf-life. The effect of UV can be more intensified if the process is coupled with other processes to bring about desired changes.

### Ozone

#### Theory

Ozone, chemically written as O_3_, contains three molecules of oxygen. It is a colorless gas with a typical odor. It is formed when molecular oxygen (O_2_) combines with singlet O. Ozone is denser in gas form than air. Ozone is a very reactive gas, and it is very much unstable and cannot be stored and needs to be produced on the spot when needed. Ozone is extensively employed as an effective antibacterial against many bacteria in food. It can be used in gas form or it can be mixed with water to form ozonated water. There are many ways by which ozone causes microbial cell death. Ozone alters the permeability of cells by damaging the microbial cell membranes. Ozone is also known to damage the structure of proteins, leading to the malfunctioning of microbial enzymes, which affects the metabolic activity and finally results in microbial cell death (192, 193).

#### Applications

Gimenez et al. (194) reported on the effectiveness of ozone against *L. monocytogenes* present in meat. Treatment of 280 mg O_3_/m^3^ for 5 h with pulse of ozone passed after 10 min for 30 min duration was effective, but an increase in the treatment time showed a change in color and oxidative damage to the lipids present in meat. Thus, to reduce the exposure time of ozone, it is combined with other treatments of food additives in order to enhance its effectiveness without any damage to food. Such studies were reported for the inactivation of *Salmonella* (195) and spoilage microorganisms (196). Ozone treatment of fruits after harvest enhances their physical, chemical, and textural properties with a reduction in the microbial load when stored in modified atmosphere packaging for 15 days (197). It is extensively used in the reduction of microbes in fruit juices (198–200). Ozone treatment is also effective in the inactivation of toxins present in food (193). There are many research reports in the literature proving the potential of ozone in the food sector, but these studies are on a laboratory scale and are not commercialized. Ozone is used in the industry for the disinfection of processing equipment. It is a very reactive molecule that reacts with many components in food, leading to undesirable changes. It also induces oxidation in food lipids; thus, it can be used in combination with other techniques. There is a need for thorough studies regarding the doses of ozone in order to reduce undesirable changes in food and to improve its acceptability. Efforts are required to increase consumer acceptability of ozone-treated food, which will force the food industry to adopt this technology to process food and market ozone-treated food.

## Conclusion

Non-thermal treatments are among the most focused research areas in the food sector due to consumer demands for safe and nutritious food free from microbes. The food product is exposed to non-thermal treatment for a very short period of time and food is treated at ambient temperature. Since the exposure time is short and the temperature is low, there are no chances of damage to heat-sensitive nutritional components in food, no damage to the food texture, and no chances of the formation of any toxic compound in food due to heat. Thus, with non-thermal treatments, consumers get fresh processed food with high nutrition and good color and flavor. But there are two sides of the coin: with advantages come some disadvantages as well. If food is exposed for a longer period or treated at a higher intensity, these non-thermal technologies may lead to some undesirable changes in food, such as oxidation of lipids and loss of color and flavor. But these technologies have many advantages compared to thermal processing. Additionally, the development of equipment to process food in bulk using non-thermal technology, understanding the proper mechanisms, development of processing standards using non-thermal treatments, and clarifying consumer myths and misunderstanding about these technologies will be helpful in the promotion of non-thermal technologies in the food sector. Once these limitations are properly overcome in a planned manner, non-thermal technologies will have a broader scope for development and commercialization in food processing industries, delivering safe and nutritious food with good color and mouthfeel to consumers.

## Author Contributions

RD: conceptualization, skeleton of the manuscript, and reviewing/editing. UA: correction of draft and editing. HJ: preparation of draft and making corrections as per suggestions. All authors contributed to the article and approved the submitted version.

## Conflict of Interest

The authors declare that the research was conducted in the absence of any commercial or financial relationships that could be construed as a potential conflict of interest.
